# Difference-frequency generation in optically poled silicon nitride waveguides

**DOI:** 10.1515/nanoph-2021-0080

**Published:** 2021-05-03

**Authors:** Ezgi Sahin, Boris Zabelich, Ozan Yakar, Edgars Nitiss, Junqiu Liu, Rui N. Wang, Tobias J. Kippenberg, Camille-Sophie Brès

**Affiliations:** Ecole Polytechnique Fédérale de Lausanne, Photonic Systems Laboratory, 1015 Lausanne, Switzerland; Ecole Polytechnique Fédérale de Lausanne, Laboratory of Photonics and Quantum Measurements, 1015 Lausanne, Switzerland

**Keywords:** all-optical poling, difference-frequency generation, integrated optics, second-order nonlinearity

## Abstract

Difference-frequency generation (DFG) is elemental for nonlinear parametric processes such as optical parametric oscillation and is instrumental for generating coherent light at long wavelengths, especially in the middle infrared. Second-order nonlinear frequency conversion processes like DFG require a second-order susceptibility *χ* ^(2)^, which is absent in centrosymmetric materials, e.g. silicon-based platforms. All-optical poling is a versatile method for inducing an effective *χ* ^(2)^ in centrosymmetric materials through periodic self-organization of charges. Such all-optically inscribed grating can compensate for the absence of the inherent second-order nonlinearity in integrated photonics platforms. Relying on this induced effective *χ* ^(2)^ in stoichiometric silicon nitride (Si_3_N_4_) waveguides, second-order nonlinear frequency conversion processes, such as second-harmonic generation, were previously demonstrated. However up to now, DFG remained out of reach. Here, we report both near- and non-degenerate DFG in all-optically poled Si_3_N_4_ waveguides. Exploiting dispersion engineering, particularly rethinking how dispersion can be leveraged to satisfy multiple processes simultaneously, we unlock nonlinear frequency conversion near 2 μm relying on all-optical poling at telecommunication wavelengths. The experimental results are in excellent agreement with theoretically predicted behaviours, validating our approach and opening the way for the design of new types of integrated sources in silicon photonics.

## 1 Introduction

Silicon nitride, with tunable material composition [1] and well-developed material processing techniques [2], has been experiencing a surge of interest for both linear and nonlinear photonics [3], [4]. Considering widely employed integrated photonic materials, stoichiometric silicon nitride (Si_3_N_4_) is free from two-photon absorption at telecommunication wavelengths as a result of its 5 eV bandgap, in contrast to silicon, and offers a Kerr nonlinearity, an order of magnitude higher than silica. Si_3_N_4_ waveguides have recently achieved optical losses as low as 1 dB/m [5], [6]; moreover, it has a wide transparency from the visible to middle infrared (mid-IR), and exhibits a weak stimulated Brillouin scattering gain [7] enabling high power handling capability. Si_3_N_4_ platform has been exploited for supercontinuum generation [8], [9], soliton microcombs [10], [11] and spectroscopy [12], [13]. Despite these advances, Si_3_N_4_ suffers from low second-order susceptibility, *χ* ^(2)^, due to its centrosymmetric nature, which inhibits three-photon mixing processes, such as second-harmonic generation (SHG), sum-frequency generation and difference-frequency generation (DFG). Scientific interest has been shifting towards investigating methods to realize *χ* ^(2)^ processes on integrated platforms. In centrosymmetric materials, forcing the symmetry breaking can induce an effective *χ* ^(2)^ to make up for the absence of the inherent second-order nonlinearity. A broader range of nonlinear optical operations in the integrated platforms is achieved through symmetry breaking on waveguide interfaces [14], [15] or through quasi-phase-matching (QPM) induced by optical [16–18] or electrical fields [19]. All-optical poling in Si_3_N_4_, first demonstrated by Billat et al. [16], does not require complex fabrication techniques or intricate designs to achieve QPM for *χ* ^(2)^ processes, making it straightforward to implement.

The availability of a *χ* ^(2)^ response in an optical waveguide can be used for DFG, which is instrumental for generating coherent light at longer wavelengths. There have been demonstrations of near-degenerated DFG in integrated platforms such as AlGaAs Bragg reflection waveguides [20], periodically poled LiNbO_3_ ion diffused waveguides [21], as well as mid-IR wavelength generation using periodically-inverted GaAs/AlGaAs waveguides [22], periodically poled Ti:LiNbO_3_ channel waveguides [23], and electrically poled LiNbO_3_ large cross section waveguides [24]. Still, the DFG process has not been presented in a typical silicon photonics material such as Si_3_N_4_.

Here, we demonstrate DFG in the all-optically poled Si_3_N_4_ waveguides. Leveraging *χ* ^(2)^ gratings inscribed with a simple telecommunication source, we show both near-degenerate and non-degenerate DFG towards the mid-IR, the latter exploiting the dispersion properties of properly tailored waveguides. The obtained conversion efficiency (CE) spectra are in good agreement with theoretical predictions, both in terms of bandwidth and strength, while the thermo-optic effect allows tunability of QPM [25]. The current value of CE is measured in the %/W scale, and our results could be further improved through more advanced dispersion engineering as to enable the use of a lower photon energy pump. This first demonstration shows a way of designing new types of integrated sources in silicon photonics.

## 2 Principle

**Figure 1:**
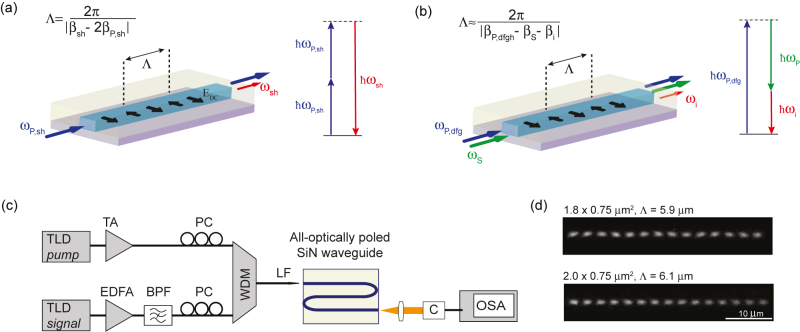
DFG in optically poled Si_3_N_4_ waveguides. (a) Grating inscription using TE polarized light. A pump wave at *ω*_P,sh_ initiates the coherent photogalvanic effect resulting in efficient SH generation from an inscribed grating with a given period Λ. (b) Illustration of DFG between pump at *ω*_P,dfg_ and signal at *ω*_s_ relying on the previously inscribed grating. The SH QPM period Λ is fixed and should also satisfy the DFG QPM condition for efficient generation of the idler wave at *ω*_i_. (c) Schematic of the DFG setup. TLD: tunable laser diode, EDFA: Erbium-doped fiber amplifier, TA: tapered amplifier, BPF: bandpass filter, PC: polarization controller, WDM: wavelength division multiplexer, LF: lensed fiber, C: collimator, OSA: optical spectrum analyser. (d) Two-photon images of the nonlinear gratings inscribed in the two waveguides used in this study. The period of the grating is indicated by two full oscillations in the grating images, as the ${\left(\chi^{\left(2\right)}\right)}^{2}$ response is measured.

To induce the $\chi_{\text{eff}}^{\left(2\right)}$ needed for DFG, the Si_3_N_4_ waveguides are first all-optically poled [18], [25]. High peak power nanosecond pump pulses are injected into the waveguide, altering the position of the charges transversly along the waveguide in a spatially periodic fashion following the coherent photogalvanic effect. The resulting periodic DC field has a periodicity Λ = 2*π*/|*β*_sh_ − 2*β*_P,sh_|, where *β*_P,sh_ and *β*_sh_ are the propagation constants at the poling pump and its second harmonic (SH), respectively (Figure 1a). $\chi_{\text{eff}}^{\left(2\right)}$ is induced and QPM between the poling pump and its SH is naturally satisfied.

The inscribed grating with the fixed period Λ, initially matched for SH generation at the poling pump wavelength, can be used for DFG provided that fundamental modes of light for the respective waveguide geometry satisfy:

(1) $$\left|\beta_{\text{sh}}-2\beta_{\text{P,sh}}|=|\beta_{\text{P,dfg}}-\beta_{\text{s}}-\beta_{\text{i}}\right|$$

where the subscripts, on the right-hand side, s, i and, P,dfg denote the signal, idler, and pump involved in the DFG process, respectively (Figure 1b).

Evidently, near-degenerate DFG where *ω*_P,dfg_ ≈ *ω*_sh_, and *ω*_s_ ≈ *ω*_P,sh_ satisfies Eq. (1) with an idler generated close to the signal (*ω*_s_ ≈ *ω*_i_) and can serve as a confirmation of the possibility to realize reverse processes in optically poled waveguides. A key method to alter the spectral range of operation for DFG, as to push it towards the mid-IR, is dispersion engineering through waveguide dimension optimization, as clearly indicated in Eq. (1). The prediction of a grating period for a specific waveguide dimension and poling wavelength, as well as the modelling of interacting wavevectors, enables the numerical optimization of the DFG process with available sources. More details on dispersion engineering can be found in the Supplementary Material. The performed work indicates the possibility of the idler generation in the region 1.9 μm using the pump near 840 nm and signal in the C-band range. Such dispersion engineering and numerical optimization within a broader search space will unlock a wider band of operation for DFG. Overall, rethinking how dispersion can be utilized as to satisfy multiple processes simultaneously represents a new way of designing mid-IR sources based on DFG while solely relying on waveguides all-optically poled using standard telecommunication laser sources.

## 3 Experiment and results

Based on the dispersion engineering simulations presented in Figure S1, we used the Si_3_N_4_ waveguides having cross-sections of 1.8 × 0.75 μm^2^ and 2.0 × 0.75 μm^2^. Both waveguides are 5.5 cm long, folded in 11 meanders, and buried in SiO_2_ cladding. They are fabricated using the photonic damascene process that enables low optical loss, high optical confinement, and dispersion engineering through geometry variation [26]. For poling, we used a tunable wavelength source operating in the telecom band. The light was shaped in 1 ns square-shaped pulses at a repetition rate of 5 MHz by a Mach–Zehnder modulator and amplified. It is then coupled to the waveguide using a lensed fiber resulting in on-chip peak power around 100 W [18]. During poling, the chip temperature is maintained at 30 °C via a PID controller, a Peltier element, and a temperature transducer. After poling, we extract values of $\chi_{\text{eff}}^{\left(2\right)}$ and the grating length *L* through a least-squares fit of the measured SHG CE spectrum throughout the C-band [25]. The CE spectra can be found in the Supplementary Material. The period Λ of the all-optically inscribed grating is theoretically calculated and is in excellent agreement with the experimentally measured ones (Figure 1d). $\chi_{\text{eff}}^{\left(2\right)}$ values are estimated in the 0.06–0.19 pm/V range, similar to previously reported values. These parameters, summarized in Table 1, are used to calculate the spectral dependence of QPM for DFG processes, as will be elaborated later in the section below.

**Table 1:** Employed waveguide characteristics.

Cross-section	1.8 × 0.75 μm^2^	2.0 × 0.75 μm^2^
Length	5.5 cm	5.5 cm
Min. bend radius	100 μm	100 μm
Loss (at 1550 nm)	4 dB/m	4 dB/m
$\chi_{\text{eff}}^{\left(2\right)}$	0.19 pm/V (TE)	0.07 pm/V (TE)
	0.06 pm/V (TM)	
*L*	2.06 cm (TE)	3.82 cm (TE)
	2.94 cm (TM)	
Λ	5.84 μm (TE)	6.06 μm (TE)
	4.77 μm (TM)	

The optical setup for DFG in poled waveguides is shown in Figure 1c. Tunable laser diodes cascaded with amplifiers are used for both pump and signal arms. The signal was filtered using a bandpass filter to reduce the noise floor and increase the visibility of the idler. Since the waveguide is birefringent and can be poled to operate in either TE or TM polarization, both pump and signal arms include a polarization controller. After combining with a WDM coupler, we couple pump and signal into the waveguide using a lensed fiber. At the output of the waveguide, the light is coupled back to a multimode fiber using a collimator and delivered to the optical spectrum analyzer.

### 3.1 Near-degenerate DFG

**Figure 2:**
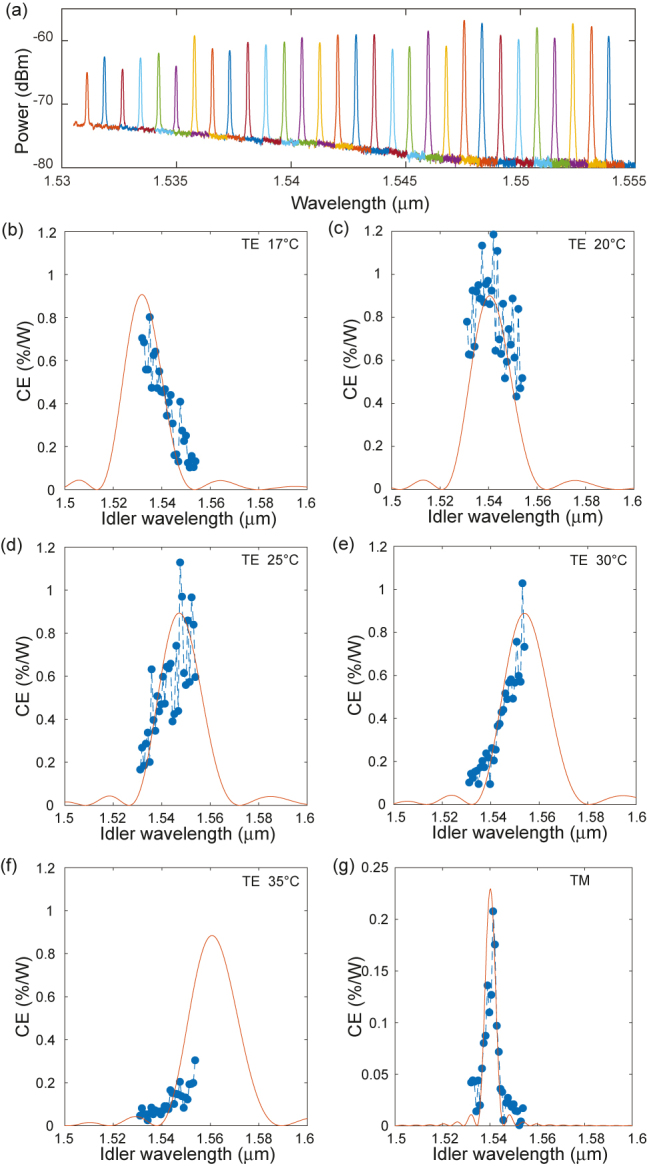
Near-degenerate DFG in optically poled 1.8 × 0.75 μm^2^ Si_3_N_4_ waveguide (a) idlers spectra generated from a 1570 nm signal and a pump tuned from 775 to 781 nm (0.2 nm step) at 25 °C for TE poling. (b–f) DFG theoretical (full lines) and measured CE (dots) as a function of idler wavelength for chip temperature of 17 °C, 20 °C, 25 °C, 30 °C and 35 °C, all TE. (g) DFG theoretical and measured CE as a function of idler wavelength for TM.

First, we investigated near-degenerate DFG using the 1.8 × 0.75 μm^2^ waveguide poled at 1555 nm. We fixed the signal at 1570 nm while we varied the pump wavelength between 775 and 781 nm (range limited by the source) as to understand and showcase the DFG behaviour according to the QPM spectral dependence induced by all-optical poling. While varying the pump wavelength, both the pump and signal coupled input powers were kept at the level of −9 dBm and 18 dBm, respectively. Figure 2a shows the spectrum of the idlers generated at *ω*_*i*_ = *ω*_P,dfg_ − *ω*_s_ for the chip temperature of 25 °C and using TE polarization.

The experimental CE is calculated based on the on-chip output powers using CE = *P*_i_/*P*_s_ *P*_P,dfg_, with *P*_i_, *P*_s_ and *P*_P,dfg_ the idler, signal, and pump powers, respectively. The insertion loss per facet is estimated to be 3 dB for telecommunication band and 6.5 dB at the pump wavelength. The theoretical DFG CE in all-optically poled waveguides can be calculated based on the phase-matching condition. The wave-vector mismatch Δ*β* is given by:

(2) $$\Delta \beta =2\pi \left(\frac{n_{\text{eff}}^{\text{P,dfg}}}{\lambda_{\text{P,dfg}}}-\frac{n_{\text{eff}}^{\text{s}}}{\lambda_{\text{s}}}-\frac{n_{\text{eff}}^{\text{i}}}{\lambda_{\text{i}}}-\frac{1}{\Lambda }\right)$$

where $n_{\text{eff}}^{\text{P,dfg}}$ , $n_{\text{eff}}^{\text{s}}$ , $n_{\text{eff}}^{\text{i}}$ , *λ*_P,dfg_, *λ*_s_, and *λ*_i_ are the effective refractive indices and wavelengths of the DFG pump, signal and idler, respectively. The CE is then calculated according to:

(3) $$\text{CE}=\frac{2\pi^{2}{\left(\chi_{\text{eff}}^{\left(2\right)}\right)}^{2}}{n_{\text{eff}}^{\text{P,dfg}}n_{\text{eff}}^{\text{s}}n_{\text{eff}}^{\text{i}}c\epsilon_{0}\lambda_{\text{i}}^{2}}\frac{L^{2}}{A_{\text{eff}}}\frac{\sin^{2}\left(\frac{\Delta \beta L}{2}\right)}{{\left(\frac{\Delta \beta L}{2}\right)}^{2}}$$

where $\chi_{\text{eff}}^{\left(2\right)}$ and *L* are extracted from the experimentally measured SHG CE, and *A*_eff_ is the effective area calculated as $A_{\text{eff}}=A_{\text{eff}}^{\text{P,dfg}}A_{\text{eff}}^{\text{s}}/A_{\text{eff}}^{\text{i}}$ using modal simulations. The theoretically expected sinc-shaped CE spectrum for the near-degenerate DFG in TE polarization has a relatively wide bandwidth owing to the small mismatch in group indices of the involved waves. The limited tuning range of the 780 nm pump source could not allow for a full characterization (e.g. see Figure 2b); therefore to circumvent this limitation, we leveraged the temperature dependence of our waveguide calculated to be Δ*λ*/Δ*T* = 0.691 nm/°C [25]. Through temperature tuning from 17 °C to 35 °C, we altered the spectral position of the CE peak and were able to scan through different sections of the theoretical curve as shown in Figures 2b–f. The theoretical and the experimental data in both peak and tails sections of the expected sinc-shaped curve are in good agreement.

For the same 1.8 × 0.75 μm^2^ waveguide, the pump, signal and idler waves exhibit more rapid change of Δ*β *with wavelength in TM polarization. In this case, the theoretically expected DFG CE spectrum has a much narrower bandwidth than that in the TE case. This trend is confirmed experimentally as seen in Figure 2g where, after poling in TM, the full CE bandwidth is covered without requiring temperature tuning.

### 3.2 Non-degenerate DFG

**Figure 3:**
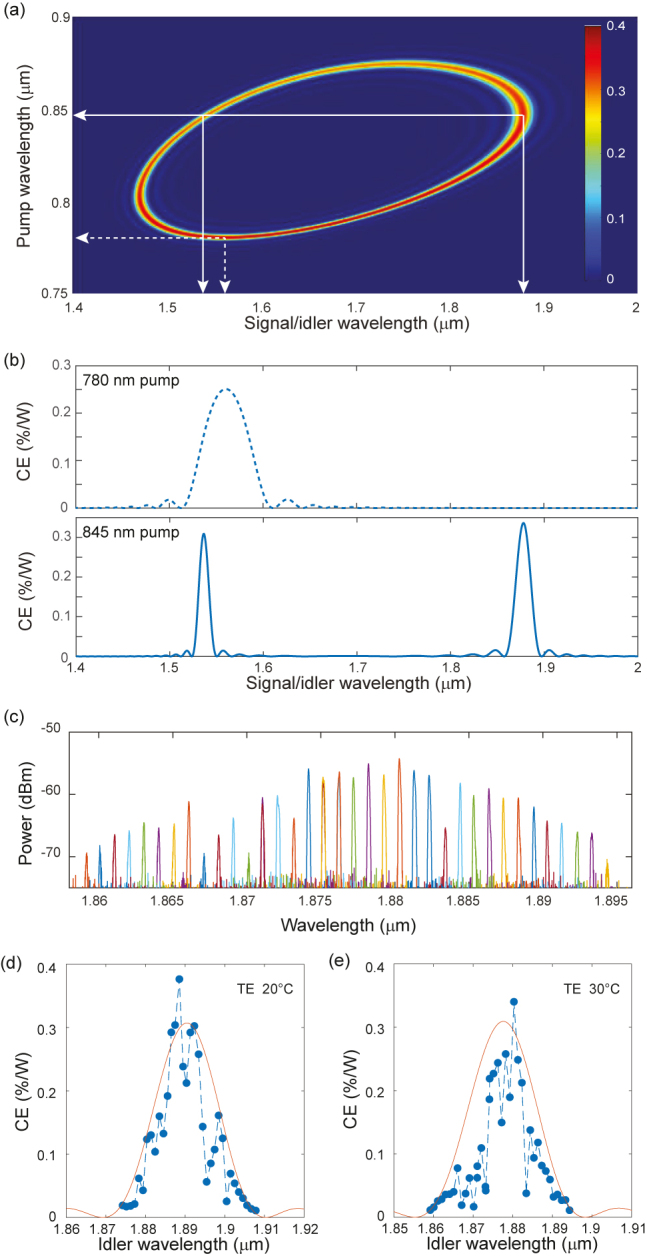
Non-degenerate DFG in all-optically poled Si_3_N_4_ waveguide with a cross-section of 2.0 × 0.75 μm^2^ (a) simulated DFG CE (in %/W) map for a 1560 nm poling wavelength showing possible QPM with a DFG pump wavelength between 780 and 880 nm. (b) CE versus signal/idler wavelength for a 780 nm (broadband near-degenerate DFG) and 845 nm (non-degenerate DFG) pump, corresponding to the dashed lines and full lines in part (a), respectively. For the 780 nm pump, the process is near-degenerate, while distinct peaks are obtained for 845 nm pump. (c) Spectra of idlers generated by mixing 1535 nm signal and pump ranging from 841 to 848 nm at 30 °C. (d and e) DFG theoretical (full lines) and measured CE (dots) as a function of idler wavelength for chip temperature of 20 °C and 30 °C, respectively.

The results shown in Figure 2 establish, for the first time, the feasibility of DFG in all-optically poled Si_3_N_4_ waveguides. The next step was to carry out thorough simulations for the expected DFG efficiency to identify QPM regions for other DFG pump wavelengths, sweeping through the design space of available waveguides, poling wavelength (limited to the C- and L-band) and DFG pump/signal sources (see Supplementary Material). The intensity map in Figure 3a shows the DFG QPM wavelengths for the waveguide with a cross-section of 2.0 × 0.75 μm^2^, where the poling wavelength is 1560 nm. We identify possible QPM with telecom signals when a DFG pump is tuned between 780 and 880 nm. In Figure 3b, we show the expected CE as a function of signal/idler wavelength for 780 and 845 nm pumps (dashed and full lines in Figure 3a, respectively). The former shows broadband near-degenerate DFG while the latter indicates non-degenerate DFG resulting in a 1.89 μm idler when the signal is in the C-band. Given the availability of a 840 nm pump, we experimentally tested this configuration.

We performed the experiment by first poling the waveguide (at 30 °C) with the 1560 nm pump as to write the grating needed for the QPM of DFG. Then the tunable pump near 840 nm was coupled with the C-band signal fixed at 1535 nm, where the coupled input powers for pump and signal are the same as in the near-degenerate DFG experiments; −9 dBm and 18 dBm. The measured idlers can be seen in Figure 3c. The CE spectra for this non-degenerate DFG, measured for two fixed temperature points (20 °C and 30 °C), are plotted in Figure 3d and e. To extract the $\chi_{\text{eff}}^{\left(2\right)}$ and *L* values of inscribed grating, we used the SHG CE measurement shown in Figure S2. Assuming that the $\chi_{\text{eff}}^{\left(2\right)}$ and *L* values do not have a significant spectral dependence since we are operating far from any material resonances, we simulated the CE spectrum using once again Eqs. (2) and (3). We can see that the simulated and the measured CE agree well. The quantitative deviations may be attributed to the possible uncertainties in temperature maintenance, slight divergences between modelled and actual waveguide dispersion, along with the inherent complexity of modelling a two-step nonlinear optical process, consisting of the initial all-optical poling stage and the DFG process.

**Figure 4:**
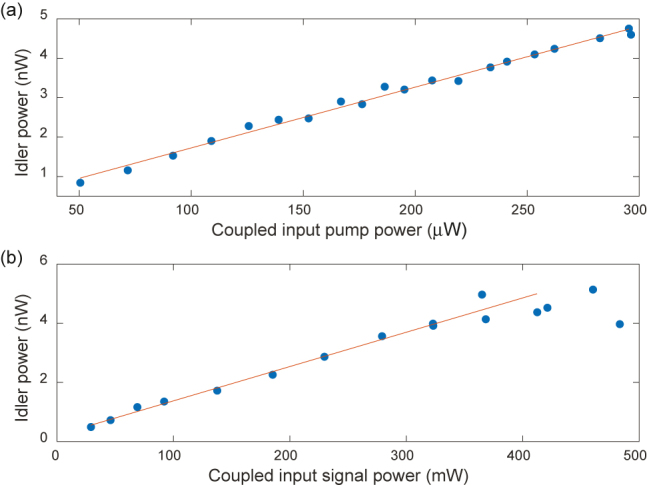
Non-degenerate DFG idler scaling with the increased power of: (a) the 840 nm pump with a CE of 0.1%/W and (b) the 1535 nm signal with a CE of 0.06%/W. Blue dots are the measurements and the lines are the linear fit as a visual guide.

Finally, to confirm the DFG process, we measured the power dependence of the output idler as a function of the coupled DFG pump and signal powers. When we changed the signal (or pump) power, we kept the pump (or signal) power equal to power levels coupled into the waveguides as in Figures 2 and 3, for consistency. Figure 4a shows the on-chip idler power as a function of the coupled input pump power. We could not increase the power beyond the measurements presented here due to the limited output power of the 840 nm laser available in our laboratory. In this low power region, the idler power scales linearly with the pump power as expected. If the pump power is further increased, we would expect to see the gradual erasure of the grating similar. The scaling of the idler power as a function of the coupled signal power is shown in Figure 4b. Once again, we can confirm that the idler power increases linearly until the coupled input signal power is around 400 mW. The linear relationship does not continue beyond this point, and we see that the measurements with the highest powers do not result in idler powers as high as theoretically predicted. By comparing the SHG CE before and after the DFG experiment, we could confirm that this saturation is not attributed to an erasure of the grating since no significant change in efficiency was observed. The reduction in CE in this case can be credited to the excessive number of signal photons compared to the pump photons [27]. When the coupled input signal power is 368 mW, for every pump photon, there is indeed more than three orders of magnitude signal photons.

To increase the power further than the operational levels used throughout the paper, we utilized the 780 nm laser with an amplifier pump source and investigated the change of idler power with that of pump in case of near-degenerate DFG. Surpassing a DFG pump power threshold may result in the reduction of the $\chi_{\text{eff}}^{\left(2\right)}$ as the inscribed grating can be erased due to the increase of mobility of the charges that make up the grating. We observed that going beyond a coupled input DFG pump power of 1.2 mW resulted in a decrease in idler power due to the grating erasure (see Supplementary Material Figure S3). While a significant advantage of all-optical poling is reconfigurability, given that the periodic spatial pattern of the charges can be modified by changing the pump wavelengths or erased via injecting high-energy photons, the use of high pump powers is limited. Such bleaching of the grating was shown to occur more rapidly for higher photon energy sources [28]. A promising way to enable coupling higher pump powers without grating erasure, and thus increase the generated idler power, is to use longer wavelength pumps. An added benefit would be that the idler wavelength could also be shifted further towards the mid-IR wavelength range.

## 4 Discussion and conclusion

The DFG process in Si_3_N_4_ has the potential to construct a building block for tunable coherent sources in a wide wavelength window, from the infrared to mid-IR, and is a feasible alternative to supercontinuum generation for extending the wavelength range to the mid-IR in typical *χ* ^(3)^ platforms. The high confinement of optical field in Si_3_N_4_ waveguides facilitates not only compact integration but also dispersion engineering and, therefore, effective frequency mixing by phase-matching. The maturity of the platform eases the integration with other devices, besides, the features like low propagation losses and high damage threshold contribute to the advantages of Si_3_N_4_ among previously demonstrated DFG processes in other platforms (Table 2). We believe that our work will break new ground for exploiting DFG in the prevailing Si_3_N_4_ platform.

**Table 2:** Comparison of various platforms for difference frequency generation in waveguides.

Ref.	Platform	*λ*_i_; *λ*_s_; *λ*_p_ (nm; nm; nm)	CE (%/W)	Idler power	Width × height	Losses (C-band)
[20]	AlGaAs	1565; 1546; 778	≈5.7 × 10^−4^	0.95 nW	4.4 × 3.6 μm^2^	8.7 dB/cm
[21]	LiNbO_3_	1597; 1539; 784	260	NA	5.5 × 1.6 μm^2^	NA
[22]	GaAs/AlGaAs	3400; 1526; 1064	3	4 nW	5 × 0.9 μm^2^	2.3 dB/cm
[23]	LiNbO_3_	2800; 3391; 1550	105	230 nW	20 × 50 μm^2^	0.1 dB/cm
[24]	LiNbO_3_	3200; 1550; 1047	40	0.26 mW	18 × 10 μm^2^	0.13 dB/cm
This work	Si_3_N_4_	1890; 1535; 846	0.3	5 nW	2 × 0.75 μm^2^	0.04 dB/cm

As a starting point, we demonstrated here near- and non-degenerate DFG on the Si_3_N_4_ platform relying on all-optical poling performed using telecommunication band sources. The $\chi_{\text{eff}}^{\left(2\right)}$ grating, optically written to automatically satisfy QPM for the C/L band poling wavelength and its SH, can be efficiently leveraged for DFG towards the mid-IR given that the adequate dispersion relation is satisfied. We show that the theoretical expectations of CE based on the simulations of the effective refractive index and the parameters extracted from the SHG characterization are in excellent agreement with the experimental measurements, validating our approach. Similar to other grating-based approaches, we show that thermo-optic tuning allows for the spectral position of the DFG QPM to be altered. Finally, we confirmed the DFG power scaling rules. Besides, the observation of DFG and SHG (see Supplemental Figure S4) indicated that both processes are simultaneously occurring regardless of the lack of the material’s intrinsic *χ* ^(2)^ and that all-optical poling is satisfactory for concurrent exploitation of multiple *χ* ^(2)^ processes. These results represent a way towards simple and compact tunable coherent light sources at large processing yields for the key operation wavelengths in the mid-IR.

## Supplementary Material

Supplementary Material Details
