# Supplementary material for: Difference-frequency generation in optically poled silicon nitride waveguides
Source: Nanophotonics. 2021 May 3;10(7):1923–30. doi: 10.1515/nanoph-2021-0080 (PMC8865395; doi:10.1515/nanoph-2021-0080)
Supplement: Supplementary file 1 — Supplementary Material Details [file j_nanoph-2021-0080_suppl.pdf]

Difference frequency generation in all-optically poled silicon nitride waveguide: supplemental document

1. DISPERSION ENGINEERING FOR DFG PROCESS

Dispersion engineering is an essential part of enabling the DFG process efficiently in an optical waveguide. Here, we assessed and controlled the dispersion of the Si_3N_4 waveguides by variation of waveguide dimensions. The final purpose of this step was to identify the optimal combination of waveguide dimensions and poling wavelength for an efficient non-degenerate DFG with available light sources.

The dispersion engineering was performed as follows. Initially, the effective refractive indices of optical modes as a function of wavelength were calculated in Si_3N_4 waveguides having different dimensions using a mode solver from COMSOL Multiphysics®. Then, the QPM period inside the waveguide was fixed by setting the poling wavelength. The normalized DFG conversion efficiency was calculated for various pump and signal wavelength pairs in a 10 mm long waveguide and plotted as shown in Fig. S1. While the numerical optimization space for an efficient DFG is quite significant given the range of waveguide dimensions, light polarization, and poling wavelength, we show the DFG conversion efficiency maps for a limited number of cases where the poling wavelength was set to 1560 nm (TE polarized) and waveguide width and height are varied in the range from 1.6 to 2.2 μm and from 0.7 to 0.8 μm, respectively. As evident from DFG conversion efficiency maps in Fig. S1, the conversion efficiency at the given pump and signal wavelengths depends critically on the waveguide dimensions. During the dispersion engineering step, we observed the feasibility of non-degenerate DFG with available pump and signal sources, for a pump wavelength around 0.84 μm and a signal wavelength within the C-band region, in a waveguide with dimensions of 2.0 x 0.75 μm^2, which was then confirmed experimentally (see main text).

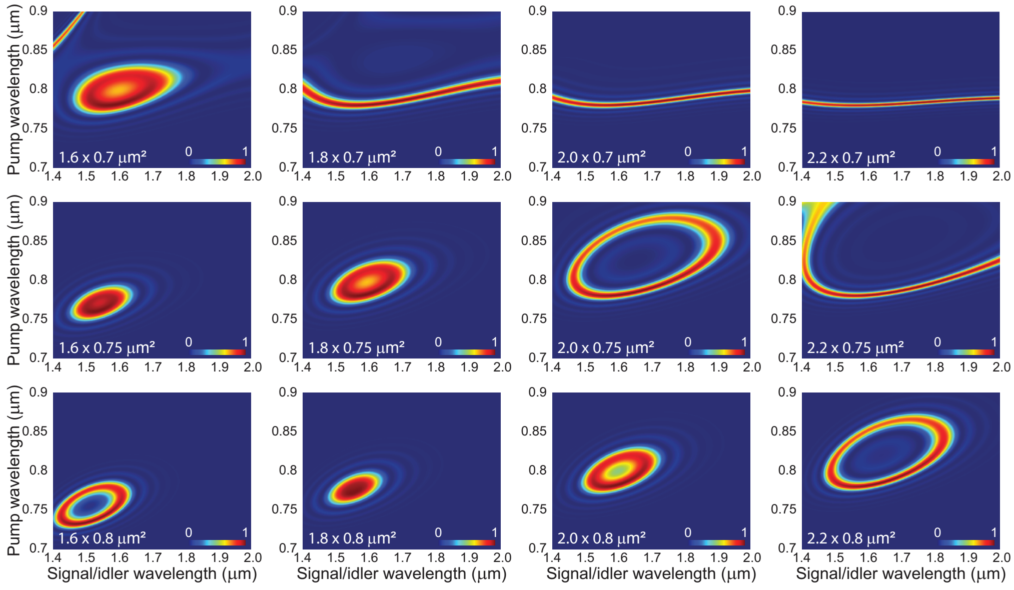

Fig. S1. Simulated DFG conversion efficiency (in a. u.) maps for different Si_3N_4 waveguide cross-sections. In each column width was fixed to 1.6, 1.8, 2.0, and 2.2 μm, respectively, while height was varied with the step of 50 nm from 0.7 to 0.8 μm in each row.

2. QPM CHARACTERIZATION BASED ON SHG

Two Si_3N_4 waveguides, with cross-sections 1.8 x 0.75 μm^2 and 2.0 x 0.75 μm^2, were all-optically poled using pump wavelengths of 1555 nm and 1560 nm, respectively. In both cases, the chip temperature was maintained at 30 °C during poling. After all-optical poling, a tunable continuous wave laser operating in the C and L bands was coupled to the waveguide and SH CE as a function of pump wavelength was measured. Such CE spectra for the 1.8 x 0.75 μm^2 and 2.0 x 0.75 μm^2 waveguides are shown in Fig. S2. Through a least-squares fit of the measured CE spectra, we extract the values of $\chi^{(2)}_{\text{eff}}$ and the grating lengths L. Mode distortions caused by the waveguide bends in the meander waveguides might contribute to the fluctuations from the expected sinc-square shape in Fig. S2 (a-c).

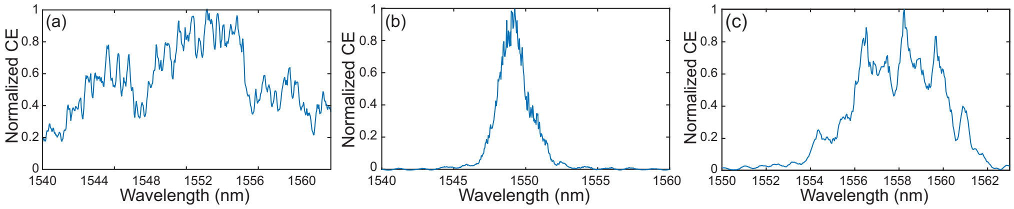

Fig. S2. Second-harmonic generation conversion efficiency spectrum measured for (a) the 1.8 x 0.75 μm^2 waveguide poled in TE polarization at 1555 nm; (b) the 1.8 x 0.75 μm^2 waveguide poled in TM polarization at 1550 nm; (c) the 2.0 x 0.75 μm^2 waveguide poled in TE polarization at 1560 nm;

3. DFG EFFICIENCY DEPENDENCE ON PUMP POWER

As discussed in the main text of the manuscript, the DFG process efficiency may decrease when the high pump power is used in the experiment. Below in Fig. S3, we display the idler power dependence on the pump power inside a waveguide having cross-section of 1.8 x 0.75 μm^2 all-optically poled at 1555 nm. As evident from the graph, the idler power initially scales linearly with pump power until a saturation followed by a decreasing due to the gradual bleaching of the grating.

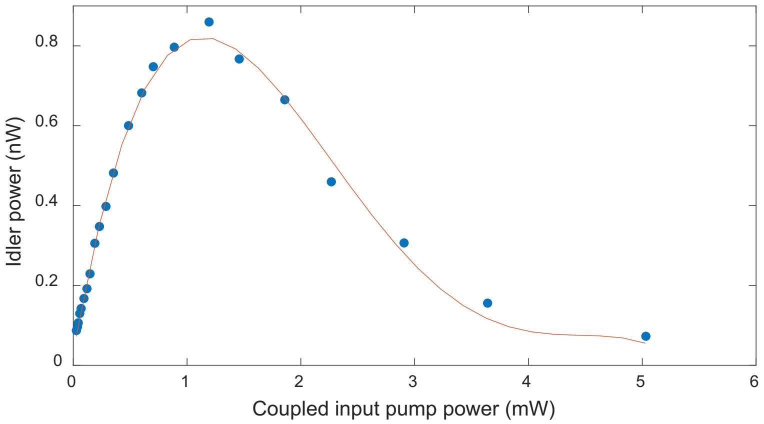

Fig. S3. DFG on-chip idler power as a function of the coupled 780 nm pump power in the 1.8 x 0.75 μm^2 waveguide poled at 1555 nm. Blue dots show the measurements, and the red line is a polynomial fit of 4th order.

4. SIMULTANEOUS $\chi^{(2)}$ PROCESSES

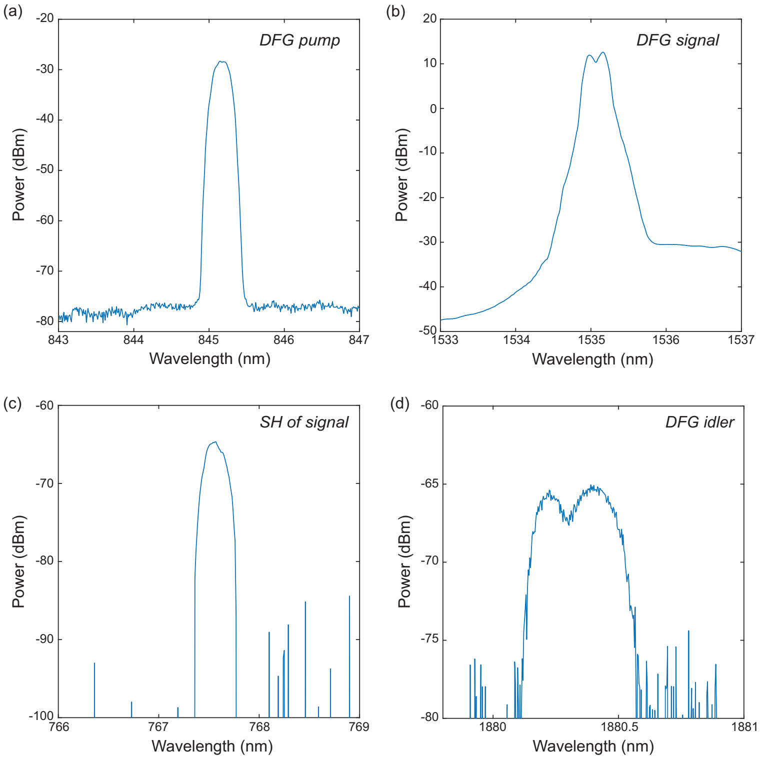

Fig. S4. Concurrent observation of DFG and SHG in the 2.0 x 0.75 μm^2 waveguide poled using TE polarized light with wavelength of 1560 nm. (a) Spectrum of the DFG pump; (b) Spectrum of the DFG signal; (c) Spectrum of the SH of the DFG signal. The efficiency is relatively low since the waveguide is optimized for SHG at the poling wavelength of 1560 nm and not the DFG signal wavelength of 1535 nm; (d) Spectrum of the DFG idler.
